# Characterization of the complete mitochondrial genome of the white grouper *Epinephelus aeneus* (Perciformes, Serranidae) and a comparative analysis with other Serranidae species

**DOI:** 10.1080/23802359.2020.1768956

**Published:** 2020-05-28

**Authors:** Seungki Lee, Dae-Sung Lee, Jong Su Yoo, Ha Yeun Song

**Affiliations:** a Biological and Genetic Resources Assessment Division, National Institute of Biological Resources, Incheon, Republic of Korea; b Department of Genetic Resources, National Marine Biodiversity Institute of Korea, Seocheon, Republic of Korea

**Keywords:** Mitochondrial genome, Perciformes, Serranidae, *Epinephelus aeneus*

## Abstract

The complete mitochondrial genome of the white grouper *Epinephelus aeneus*, which belongs to the family Serranidae, was determined. The complete mitochondrial genome measured 16,578 bp in length and consisted of 13 protein-coding genes, 22 tRNA genes, 2 rRNA genes, and a control region. The mitochondrial gene arrangement of *E. aeneus* was typical of vertebrates. Phylogenetic analysis conducted using the mitochondrial genomes of 13 related species showed that *E. aeneus* clustered with other Serranidae species. This mitochondrial genome provides an important resource for addressing taxonomic issues and developing conservation strategies.

The white grouper *Epinephelus aeneus* (Perciformes, Serranidae) is a marine fish that inhabits the southern Mediterranean Sea and the subtropical eastern Atlantic Ocean. This species is a commercially important fish in the markets of the Mediterranean and the west coast of Africa. However, there has been a massive decline in this species population over the past 30 years due to overfishing and habitat destruction (Meissa et al. 2013; Ndiaye et al. 2013). In the present study, we determined the complete mitochondrial DNA sequence of *E. aeneus* and compared it with those of other species of Serranidae.

*Epinephelus aeneus* specimen was collected from the eastern Atlantic Ocean (8.30 N, 14.00 W). Total genomic DNA that was extracted from the specimen tissue has been deposited at the National Marine Biodiversity Institute of Korea (Voucher No. MABIK0001997). The mitogenome was sequenced using the Illumina Hiseq 4000 sequencing platform (Illumina, San Diego, CA) and assembled with *SOAPdenovo* at Macrogen Inc. (Korea). The complete mitochondrial genome was annotated using MacClade ver. 4.08 (http://macclade.org/macclade) (Maddison and Maddison, 2005) and tRNAscan-SE ver. 2.0 (http://lowelab.ucsc.edu/tRNAscan-SE) (Lowe and Chan 2016).

The complete mitochondrial genome of *E. aeneus* (GenBank accession no. LC545417) measured 16,578 bp in length and consisted of 13 protein-coding genes, 22 tRNA genes, 2 rRNA genes, and a control region. The overall base composition was 29.18% A, 28.19% C, 15.52% G, and 27.11% T. Similar to the mitogenomes of other vertebrates, the AT content was higher than the GC content (Saccone et al. 1999). The *12S rRNA* (953 bp) and *16S rRNA* genes (1699 bp) were located between *tRNA^Phe^* and *tRNA^Val^* and between *tRNA^Val^* and *tRNA^Leu(UUR)^*, respectively. Of the 13 protein-coding genes, 11 started with ATG, the exception being *COI* and *ATP6*, which started with GTG. The stop codons of the protein-coding genes were TAA (*ND1*, *COI*, *ATP8*, *ND4L*, and *ND5*), T (*ND2*, *COII*, *ND3*, *ND4*, and *Cytb*), TA (*ATP6* and *COIII*), and TAG (*ND6*). A control region (892 bp) was located between *tRNA^Pro^* and *tRNA^Phe^*.

Phylogenetic trees were constructed using the maximum likelihood method with 1000 replicates using the MEGA 7.0 software (Kumar et al. 2016). The phylogenetic trees of the newly sequenced genome were compared with the mitochondrial genome sequences of 13 other complete Serranidae species acquired from the National Center for Biotechnology Information. It was confirmed that *E. aeneus* clustered with other Serranidae species (Figure 1). This mitochondrial genome provides an important resource for addressing taxonomic issues and developing conservation strategies.

**Figure 1.**
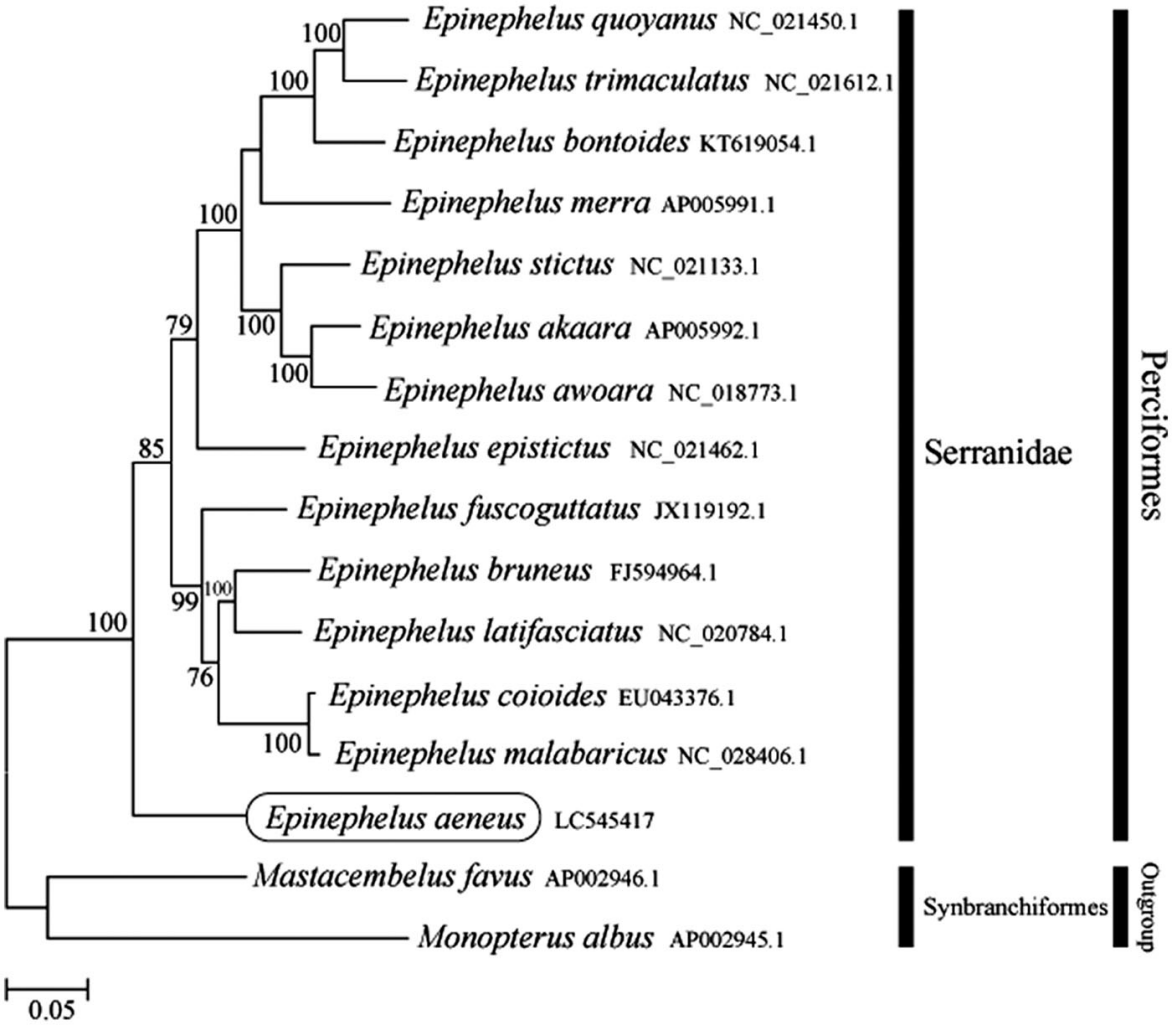
Phylogenetic position of *Epinephelus aeneus* based on comparison with the complete mitochondrial genome sequences of 13 other Serranidae species. The analysis was performed using the MEGA 7.0 software. The accession number for each species is indicated after the scientific name.

## Data Availability

The data that support the findings of this study are openly available in the DNA Data Bank of Japan (accession no. LC545417) at https://www.ddbj.nig.ac.jp.
